# On managing complex adaptive systems motivated by biosystems application to infections

**DOI:** 10.1186/1753-4631-3-11

**Published:** 2009-10-12

**Authors:** AS Hegazi, AH Hashish, E Ahmed

**Affiliations:** 1 Mathematics department, Faculty of Science, Mansoura 35516, Egypt; 2 Physics department, Faculty of Science, Mansoura 35516, Egypt

## Abstract

Many attempts to control Complex adaptive systems (CAS) have failed. Here we try to learn from biosystems to derive some principles for CAS management. An application to managing infections is given.

## Complex adaptive systems (CAS) [1,2]

Complex adaptive systems are special cases of complex systems. They are *complex *in that they are diverse and made up of multiple interconnected elements and *adaptive *in that they have the capacity to change and learn from experience.

i.1) CAS consists of adaptive (capable of learning) entities. Some properties of such systems cannot be understood just by studying the constituents. They are called emergent properties [3,4].

i.2) Examples of CAS include the brain where the emergent property is cognition which is a property of the brain not of individual cells. We propose that IS is a CAS with tolerance as an emergent property since tolerance is a property of IS as a whole not individual B or T or macrophage etc.... cells. In fact almost every biological, social and financial system is a CAS.

i.3) Emergent properties imply that reductions approach is not suitable for CAS. Hence these systems have to be studied as a whole in addition to the reductionest approach. It is crucial to see that both approaches complement each other and are not contradictory [5].

i.4) CAS have some generic properties e.g. they are open (connected to other systems). Interactions in CAS are nonlinear hence long range predictions in CAS are highly unlikely. Typically they have a network (e.g. evolving network) structure. Optimization in such systems is multiobjective. Control of most CAS is difficult, it should be targeted and integrated i.e. using diverse approaches not just one. Most CAS show delay and memory behavior. Spatial effects should not be neglected in many CAS. Most, but not all, CAS are decentralized.

i.5) CAS should be studied as a whole in addition to studying its constituents. Hence in vivo experiments are crucial to understand them.

i.6) CAS are difficult to control for the following reasons: They are nonlinear. They are open. Delay is a generic property of CAS. Some failure cases in controlling CAS are the Measles-Mumps Rubella (MMR) vaccination and the change of fish type in Lake Victoria [2]. Therefore in the next sections we try to learn from some CAS systems to derive some principles for "managing" CAS.

By management we mean taking decisions with as least bad side effects as possible. And if bad side effects occurred then decisions are taken to remove or contain them as best as possible.

## Optimization in Complex adaptive systems

Some of the basic concepts for optimization in CAS are:

ii.1) Multi-objective optimization [6] especially metaheuristics i.e. methods which contain random element and which give a good approximate solution to the optimization problem within a reasonable time.

ii.2) Game theory specially evolutionary game theory [7]. In some cases it is important to include the evolution of strategies or players c.f. the immune system deals with almost continuously evolving antigen e.g. bacteria, viruses or macroparasites.

ii.3) Fractals and networks which play a crucial role in nonlocal spread of effects e.g. infections.

ii.4) Collective animal behavior. Some of its principles include [8]: Non-selfish key individuals, positive and negative feed back, diversity and threshold responses.

## Conclusions and application to infection

From the above results we conclude the following:

iii.1) CAS are extremely difficult to control hence managing CAS may be a more feasible goal than controlling them.

iii.2) Diverse and integrated approaches to the problem are crucial. In may cases single approaches to the problem may lead to counter-productive results. Also this increases the robustness of the management.

iii.3) The existence of non-selfish key individuals is necessary to solve many problems.

iii.4) The goals should be multi-objective and only guide lines (metaheuristic) approaches should be adopted. One cannot attempt exact determination or control in nonlinear and open systems. Hence no detailed plans should be laid out, instead guide lines may be more realistic.

iii.5) Small world network structure should be followed hence a kind of hierarchical network with short cuts can significantly improve the efficiency of the system [9].

iii.6) Feedback hence adaptive management is expected to be more efficient than decisions taken once and for all. Also considering the system as game theory with evolving strategies or opponents may help.

iii.7) Emergent problems "messes" happen and will happen.

Now the above conclusions are applied to an overview of infection management. We propose to call such problems "emergent problems"[10]. An emergent problem is characterized by:

1) It has several reasons not just one.

2) It cannot be solved locally (e.g. by one country) hence "**collective efforts**" are needed.

3) It needs a long time to be solved hence evolution in solution strategies has to be taken into consideration.

Collective efforts needed to solve such emergent problems require large scale cooperation. Humans are known not to be good in cooperation. In fact a core problem in most of the above problems is the tragedy of commons [11] which simply states that in the case of finite resources e.g. drugs (which is always the case) a conflict exists between individual's interest and the group's (common) interest e.g. rich and poorer countries. However it is known that for such cooperation to exist a feeling of imminent danger has to exist [12].

Therefore early preparations to deal with such problems are not expected to be as efficient as should be.

It is clear that the absence of non-selfish key individuals is a key reason for the inability to solve this problem.

However some clusters of cooperators do exist. This should be accompanied by retaliation against non-cooperators to lower their benefits from defection (c.f. prisoner's dilemma game [7]).

Some benefits from our discussion are:

i) Diverse approaches to the problem is highly desirable e.g. using multi-drugs to reduce the danger of drug resistance (c.f. the sole dependence on Tami flu for avian influenza).

ii) The network (long range) effects are crucial in many infections e.g. foot and mouth disease, SARS, Avian flu and H1N1 flu.

iii) We should take into account the evolution of antigens (c.f. the new strains of Tuberculosis).

## Competing interests

The authors declare that they have no competing interests.

## Authors' contributions

All three authors contributed equally to this article. All authors read and approved the final manuscript.
